# Correction: Epigenetic, psychological, and EEG changes after a 1-week retreat based on mindfulness and compassion for stress reduction in healthy adults: Study protocol of a cross-over randomized controlled trial

**DOI:** 10.1371/journal.pone.0297812

**Published:** 2024-01-23

**Authors:** Gustavo G. Diez, Ignacio Martin-Subero, Rosaria M. Zangri, Marta Kulis, Catherine Andreu, Ivan Blanco, Pablo Roca, Pablo Cuesta, Carola García, Jesús Garzón, Carlos Herradón, Miguel Riutort, Shishir Baliyan, César Venero, Carmelo Vázquez

The affiliation for the seventh author is incorrect. The correct affiliations are not indicated. Pablo Roca is not affiliated with #1 and #6 but with: Faculty of Health Sciences, Universidad Villanueva, Spain and Valencian International University, Valencia, Spain.
